# Paraphrenia phantastica. A case report

**DOI:** 10.1192/j.eurpsy.2021.2098

**Published:** 2021-08-13

**Authors:** A. Hernández Mata, A. Sotillos Gómez, P. Marco Coscujuela, R. Pinilla Zulueta

**Affiliations:** Psychiatry, Hospital Universitario de Getafe, Getafe, Spain

**Keywords:** Paraphrenia

## Abstract

**Introduction:**

Paraphrenia is a classic diagnostic entity characterized by an insidious development of a vivid and exuberant delusional system, more or less systematized, hallucinations and confabulations.

**Objectives:**

Increase knowledge about paraphrenia, a classic diagnosis that no longer appears on international classifications.

**Methods:**

Extensive research on the historical path of the paraphrenia diagnostic entity was carried out. Patient’s data is obtained from medical history and psychiatric interviews done during her hospitalizations.

**Results:**

68 year-old patient attended the hospital emergency service due to a demonic possession delusion that emerged when she was 44 year-old, when she first consulted a psychiatrist because she believed someone introduced the demon inside her body. She described kinesthetic hallucinations as “movements of her brain” and an intense headache, both originated by the demon; as well as other types of hallucinations and confabulations. However, there was no deterioration in her personaliy or her intellectual capacity, as it could have been seen in a case of schizophrenia. This clinical case is considered a paraphrenia phantastica as it presents the typical features raised by the classic authors (mainly Henry Ey): paralogical thought dominance, megalomania, confabulation and integrity of relation with reality.

**Conclusions:**

Current internacional classifications do not consider paraphrenia as a differentiated diagnostic entity, as it also occurs with other classical entities. This causes a loss of important tools that would achieve a better approach to the patient’s condition.

**Disclosure:**

No significant relationships.

